# A self-assessment study of procedural skills of doctors in peri-urban district hospitals of Gauteng, South Africa

**DOI:** 10.4102/phcfm.v11i1.1975

**Published:** 2019-05-28

**Authors:** Neetha J. Erumeda, Ian D. Couper, Leena S. Thomas

**Affiliations:** 1 Ekurhuleni Health District Services, Gauteng Department of Health, Germiston, South Africa; 2 Department of Family Medicine, University of the Witwatersrand, Johannesburg, South Africa; 3 Ukwanda Centre for Rural Health, Stellenbosch University, Stellenbosch, South Africa; 4 Department of Community Medicine, University of the Witwatersrand, Johannesburg, South Africa

**Keywords:** generalist doctors, procedural skills, self-reported competence, peri-urban district hospital, family medicine training

## Abstract

**Background:**

Several studies have been carried out on procedural skills of doctors in district hospitals in rural South Africa. However, there is insufficient information about skills of doctors in peri-urban district hospitals. This paper attempts to supplement this vital information.

**Aim:**

The aim of the study was to determine self-reported levels of competence in procedural skills of doctors in peri-urban district hospitals and to assess factors influencing this.

**Setting:**

The study was undertaken in three district hospitals in two health districts of Gauteng Province.

**Methods:**

A cross-sectional descriptive study using a self-administered questionnaire was undertaken in three district hospitals in two health districts of Gauteng Province. The questionnaire assessed procedural skills based on district health service delivery requirements for doctors in district hospitals using a modified skill set developed for family medicine training in South Africa.

**Results:**

There was a wide range of self-reported competence and experience among doctors for various skill sets. Doctors were generally more competent for procedures in general surgery, medicine, orthopaedics, obstetrics and gynaecology and paediatrics than anaesthesia, ear, nose and throat and ophthalmology. There were statistically significant associations between age and overall anaesthetic competence (*p* = 0.03); gender and overall competence in surgery (*p* = 0.03), orthopaedics (*p* = 0.02) and urology (*p* = 0.005); years of experience and overall competence in dermatology skills; current hospital and overall competence in anaesthesia (*p* = 0.01), obstetrics and gynaecology (*p* = 0.015) and dermatology skills (*p* = 0.01).

**Conclusion:**

This was one of the first studies to look at self-reported procedural competence of doctors in a peri-urban setting in South Africa. The results highlight the need for regular skills audits, standardised training and updating of skills of doctors in district hospitals.

## Introduction

According to the World Health Organisation (WHO) model of effective health systems, developing the right cadres of health workers with an appropriate skills mix is an important consideration for any health system.^1^ As South Africa transforms its health system towards Universal Health Coverage (UHC),^2^ a key consideration is the re-organisation of health services and the health workers required for this. This is further elaborated in the White Paper on the National Health Insurance scheme (NHI),^3^ seen as a vehicle for UHC elaborating on the services to be rendered by health districts.

According to WHO, the district health system is defined as:

a network of primary care health facilities that deliver a comprehensive range of promotive, preventive and curative health care services to a defined population with active participation of the community; under the supervision of a district hospital and district health management team.^4^

In the South African setting, a district hospital is the first-level referral hospital for patients from community-based health services.^5^ District hospitals are classified as rural or urban depending on their location. Peri-urban hospitals are those that are in the peripheries of urban areas. District hospitals also function as a gateway for specialised care.^5^ Generalist doctors in the context of this paper are medical officers without any postgraduate training in family medicine. Most doctors in district hospital settings are generalists, providing holistic care and clinical procedures as outlined in the district hospital service-level package.^5,6,7^ The family physicians are medical specialists with postgraduate training in family medicine and are considered as expert generalists as they fulfil roles such as care provider, competent clinician and a capacity builder.^6,8^

District health services in rural settings often have greater needs for clinical procedures, hence more need for skills in doctors in that context. Many studies have identified the skills gap of doctors working in rural settings.^9,10,11,12,13,14,15^ According to Peters et al., the role and scope of practice of general practitioners (GPs) in urban and peri-urban district hospitals in South Africa is wider and not as well documented.^16^ Peters and colleagues also showed the need of medical officers to be competent generalists who are able to deal with primary care, trauma and emergency care skills, and for adequate surgical skills to perform obstetric, orthopaedic and other surgical procedures.^16^ A study conducted in district hospitals of the Western Cape showed that the perceived competence ratings in emergency and trauma, inpatient and outpatient services were higher in older and more experienced practitioners.^11^ Their knowledge varied considerably according to their education, training, previous work experiences and the context (rural or urban) of the district hospital.^11^

In South Africa, family physicians play an important role in the training and education of family medicine registrars, generalist medical officers, medical students, interns and clinical associates in district hospitals.^17^ Because of inadequate numbers of family physicians in district health services, the generalist medical doctors often function as in-service trainers for these students. These generalist doctors have many years of experience in a particular discipline, and therefore the assumption is that they are able to contribute significantly to the training.^7^ Existing generalists in district hospitals may be competent in specific clinical areas, and often because of long years of service, their skill competency may be more than newly qualified family physicians, thus making them a valuable potential teaching resource.^8^ The procedural skills of these generalist doctors and family physicians should be aligned to service delivery contexts in South Africa, both urban and rural, to respond to increasing health service demands. Although full-time family medicine training has evolved since this study was initially conducted, and continues to evolve, there are still inadequate numbers of family physicians in South African district hospitals.^18^ Hence clinical procedures in district hospitals are still mostly done by generalist medical officers, with these hospitals continuing as training sites for postgraduate family medicine registrars, undergraduate medical students, nurses and mid-level health workers. This study, therefore, provides an important baseline for district health service strengthening in South Africa.

The required skill set for qualified family physicians used in the current training of family medicine registrars and medical officers was developed through a national Delphi process,^19^ and then this was further refined by the eight medical schools in South Africa. The skill set is inclusive of the requirements of the district hospital service package for procedures to be done in district hospitals.^5^

Internationally, many developed countries such as the United Kingdom,^20^ the United States^21^ and Canada^22,23,24^ are following a similar trend of developing context-specific skill sets for their family medicine residency training programmes in rural and urban settings. According to Kalu et al., there is a suggestion that there should be a separate skills list for generalist doctors working in urban and rural settings in South Africa.^25^ There may, therefore, be a greater need for doctors with context-specific procedural skills in anaesthesia, obstetrics and gynaecology (O&G) and surgery for GPs working in rural and peri-urban district hospitals in South Africa to meet the current context requirements.

Few South African studies have explored the procedural skills of doctors in urban and peri-urban district hospital settings. Hence the aim of this study was to assess competence in procedural skills and the factors that influence it among doctors working in district hospitals in Gauteng Province. It is anticipated that the findings of this study, set in an urban area, in one of the most populated provinces of South Africa, will add to the body of local knowledge and inputs to the current deliberations on the role of GPs and family physicians within the proposed NHI.

## Methods

### Study design

A cross-sectional descriptive study was conducted during the period October and November 2009 using a self-assessment questionnaire.

### Setting

Three peri-urban district hospitals in Gauteng Province were purposively selected. One of the hospitals, District Hospital A, had a service delivery model where all doctors were expected to be competent in all clinical disciplines, and were, therefore, rotated in all units. In the other two, hospitals B and C, health services were divided according to the various disciplines or units, and doctors could choose to work in only one, for example, outpatient department (OPD), maternity or theatre.

### Study population and sampling strategy

All doctors working in the identified three district hospitals were invited to participate in the study. The number of doctors per district hospital ranged from 15 to 25 doctors. The study population included all categories of doctors working in the district hospital: community service doctors, GPs, medical officers, registrars in family medicine and family physicians.^6,8^

### Data collection

Data were collected using a structured self-administered questionnaire. The questionnaire was distributed through the office of the hospital clinical managers, who assisted in distributing and collecting the completed questionnaires. The questionnaire was developed using the national department of health service delivery package for district hospitals^5^ and the modified procedural skills list developed by Mash et al. for family medicine registrar training in South Africa^19^; it was further refined for the local study context. The tool was piloted in one of the study sites; as there were no major challenges or changes to the tool, these results were also incorporated into the main study. The questionnaire had two parts: the first part included questions about the general characteristics of the participants, such as ‘In which department or discipline do you spend most of your time?’, ‘Where did you train for your undergraduate medical degree?’, ‘Have you completed any postgraduate family medicine training in South Africa?’ and ‘If no, are you currently enrolled for family medicine training?’ The second part required doctors to rate their own competency against a list of skills, categorised in 10 domains. *Competence* was interpreted based on self-reported confidence of the doctor in performing a procedure, with ratings from 0 to 3. The response ‘0’ indicated unfamiliarity with the procedure; ‘1’ indicated ability to explain the procedure to a patient (theoretical knowledge) but not able to perform it; ‘2’ indicated ability to perform a procedure with support and ‘3’ indicated the ability to perform a procedure independently.

Doctors who rated themselves as ‘3’ on any item were then asked to indicate their *experience* by providing the approximate number of times they had performed the respective procedure independently, by selecting one of three options: less than 5 times was categorised as 3A, 5–10 as 3B and more than 10 as 3C. For example, if Dr X had performed endotracheal intubation independently and had done such procedures 8 times, then Dr X would score as ‘3B’. If Dr Y had performed a pleural tap with support, then Dr Y would score a ‘2’.

### Data analysis

Data were collated using Epi Info version 3.5.1 software.^26^ Firstly, descriptive statistics in the form of frequencies and percentages were used to determine the competence and experience in *each* of the procedures. If more than 75% of doctors were able to perform a procedure independently, then that procedure was categorised as highly competent; if 50% – 75%, then that was classified as a competent procedure and if less than 50%, then that was reported as not a competent procedure. Secondly, overall levels of competence in each *group* of procedural skills were determined. Doctors were categorised into overall not competent, competent or highly competent based on the number of procedures they could do independently in *each clinical discipline*. Associations between overall competence and categorical variables such as age, gender, years of experience, clinical discipline where most time was spent, current hospital, undergraduate training and postgraduate family medicine training were determined using Chi-square and Fischer’s exact tests. A *p-*value of less than 0.05 was considered statistically significant.

### Ethical considerations

Ethical approval for this study was obtained from the Human Research Ethics Committee of the University of the Witwatersrand (M070202) and the Gauteng Provincial Department of Health.

## Results

There were 70 doctors eligible for the study, with 59 completing the questionnaires – an 84% response rate (Table 1). Sixty-three per cent were males and 41% of the participants belong to the age group 35–44 years. Twenty-eight participants (48.3%) had more than 10 years of experience. A majority of participants (57.6%) spent most of their time in either OPD or casualty. Just more than half (51%) of the participants received their undergraduate medical training outside of South Africa, namely, the Congo (60%); Cuba (10%); India, Poland and Nigeria (6.7% each) and Uganda, Belgium and Bulgaria (3.3% each).

**TABLE 1:** General characteristics of study participants.

Characteristics	Frequency	
	*n*	%
**Age (years)**		
25–34	16	30.5
35–44	21	40.7
45–54	15	17.0
55 and above	7	11.9
**Gender**		
Male	37	63.0
Female	22	37.0
**Years of experience**		
2 years or less	3	5.2
3–5 years	17	29.3
6–10 years	10	17.2
Above 10 years	28	48.3
**Discipline in which most time is spent**		
Casualty	17	28.8
OPD/General practice/Family medicine	17	28.8
Medicine	8	13.6
Maternity	8	13.6
Surgery	3	15.1
HIV clinic	3	15.1
Psychiatry	1	1.7
Anaesthesia	1	1.7
ENT	1	1.7
**Place of undergraduate training**		
South Africa	29	49.0
Outside South Africa	30	51.0
**Postgraduate family medicine training (part-time)**		
No	49	83.1
Yes	10	17.0
**Current family medicine training (full-time)**		
No	54	91.5
Yes	5	8.5
**Current hospital**		
Hospital A	15	25.4
Hospital B	24	40.7
Hospital C	20	33.9

*n* = 59.

OPD, outpatient department; HIV, human immunodeficiency viruses; ENT, ear, nose and throat.

The results showed that the doctors expressed varying level of competence and experience in different procedures (Table 2). Procedures in each discipline were grouped based on the percentage of doctors with the ability to do the procedures independently (Tables 3–5). The participants reported being highly competent in doing procedures such as lumbar punctures (94.9%), inter-costal drains (93.2%) and endotracheal intubations (86.2%), but not competent in procedures such as the intrauterine contraceptive device (40.7%), appendicectomy (37.3%) and cricothyroidotomy (11.0%). Most of these participants (Table 2) reported experience of doing procedures more than 10 times with highly competent procedures compared to not competent procedures such as cricothyroidotomy where the doctors reported unfamiliarity with the procedure (32.0%).

**TABLE 2:** Results of competence and experience in procedural skills among some procedures in various disciplines.

Procedural skills	Total†	Competence								Experience (*n*)		
		Unfamiliar with procedure (0)		Able to explain to patient (1)		Able to do the procedure with support (2)		Able to do the procedure independently		3A (< 5)	3B (5–10)	3C (> 10)
		*n*	%	*n*	%	*n*	%	*n*	%			
Pleural tap	59	1	1.7	0	0.0	1	1.7	5	96.6	1	1	52
Lumbar puncture	59	1	1.7	0	0.0	2	3.4	56	94.9	1	1	52
Intercostal drain insertion	59	1	1.7	1	1.7	2	3.4	55	93.2	1	2	51
Endotracheal intubation	58	2	3.4	0	0.0	6	10.3	50	86.2	1	3	44
Caesarean section	57	2	3.5	5	8.8	8	14.0	42	73.7	1	0	40
Spinal anaesthesia	57	10	17.5	4	7.0	8	14.0	35	61.4	0	1	32
General anaesthesia	54	12	22.2	5	9.3	11	20.4	26	48.1	0	1	23
Insertion of IUCD	59	8	13.6	16	27.1	11	18.6	24	40.7	1	2	20
Appendicectomy	59	9	15.3	7	11.9	21	35.6	22	37.3	6	2	14
Cricothyroidotomy	57	18	32.0	19	33.0	14	25.0	6	11.0	4	1	1
Tracheostomy	59	15	25.4	23	39.0	13	22.0	8	13.6	3	0	4

IUCD, intrauterine contraceptive device.

† , *n* = 59.

**TABLE 3:** Procedural skills in which doctors rated themselves as highly competent.

List of procedural skills in which doctors rated themselves as highly competent (75% and above)	No. of doctors who could perform the procedure independently (*n* = 59)	
	*n*	%
Intravenous infusion (paediatrics)	56	100.0
Pleural tap	57	96.6
Lumbar puncture (adult)	56	94.9
I and D abscesses	55	94.8
Insertion of nasogastric tube	55	94.8
Urethral catheterisation	55	94.8
Intercostal drain insertion	55	93.2
Nasal packing	55	93.2
Normal vaginal delivery	54	93.0
Application of POP	54	91.5
Lumbar puncture (paediatrics)	53	93.0
Ear syringing	53	89.8
Immobilisation of fractures	53	89.8
Evacuation of uterus	51	86.4
Removal of foreign body (ENT)	51	86.4
Bartholin’s abscess	50	86.2
Endotracheal intubation	50	86.2
Episiotomy and suturing	50	84.7
Manual removal of placenta	49	84.5
Papanicolou smear	49	84.5
Reduction of dislocation	47	79.7
Excision of bumps and lumps	45	78.9
Debridement of wounds	44	77.2
Suprapubic catheterisation	44	77.2
Reduction of paraphimosis	44	75.9
Removal of foreign body eye	44	75.9
Ring block	43	78.2
Suprapubic bladder puncture	42	76.4

POP, Plaster of Paris; ENT, ear, nose and throat.

**TABLE 4:** Procedural skills in which doctors rated themselves as competent.

List of procedural skills in which doctors rated themselves as competent (50% to < 75%)	No. of doctors who could perform the procedure independently (*n* = 59)	
	*n*	%
Caesarean section	42	73.7
Close reduction of fractures	42	71.2
Circumcision	41	70.7
Gastric washout	41	69.5
Application of traction	38	65.5
Cauterisation of warts	36	64.3
Obstetric ultrasound	37	63.8
Skin biopsy	36	63.2
Umbilical vein catheterisation	36	63.2
Assisted breech delivery	36	62.1
Excision and incision biopsy	36	62.1
Laparotomy for ruptured ectopic	36	62.1
Repair of third degree tear	36	62.1
Spinal anaesthesia	35	61.4
Tubal ligation	35	61.4
Insertion of central venous line	35	59.3
Repair of lacerated eyelid	34	59.0
Paronychia drainage	33	58.6
Amputation of digits	33	58.6
Intrabursal/articular injections	32	56.1
Hydrocoele drainage	31	54.4
Aspiration of breast cyst	31	52.5
Excision of in growing toenail	30	51.0
Drainage of perianal haematoma	29	50.9

**TABLE 5:** Procedural skills in which doctors rated themselves as not competent.

List of procedural skills in which doctors rated themselves as not competent (less than 50%)	No. of doctors who could perform the procedure independently (*n* = 59)	
	*n*	%
General anaesthesia	26	48.1
Intraosseous infusion	26	46.4
Injection of keloids	26	44.8
Posterior colpopuncture	24	42.1
Insertion of IUCD	24	40.7
Endometrial biopsy	22	39.3
Appendicectomy	22	37.3
Skin graft	19	32.8
Venous cutdown	18	30.5
Indirect laryngoscopy	15	25.9
Proctoscopy	14	24.6
I and D Meibomian cyst	14	24.6
Pleural biopsy	14	24.0
Bier’s block	8	16.7
Epidural anaesthesia	8	14.0
Vasectomy	8	13.8
Tracheostomy	8	13.6
Cricothyroidotomy	6	11.0
Tonometry	4	6.9

IUCD, Intrauterine contraceptive device.

The participants reported being competent in performing procedures such as caesarean sections (C/S) (73.7%) and spinal anaesthesia (61.4%) (Table 3). The participants reported not being competent in procedures such as venous cutdown (30.5%), vasectomy (13.8%) and pleural biopsy (Table 5).

Comparisons were performed using Chi-square and Fisher’s exact tests between total scores of overall competence of a doctor in each set of procedures in one discipline and the main variables such as age, gender, years of experience, place of undergraduate study, discipline where they spent most of the time, family medicine training and the current hospital of employment (Table 6). Significant associations were found between age and overall anaesthetic competence (*p* = 0.03), as well as male gender and greater overall competence in general surgical skills (*p* = 0.03), orthopaedic skills (*p* = 0.02) and urology skills (*p* = 0.005), and years of experience and overall competence in dermatology skills. There were also significant associations between the current hospital and overall competence in anaesthesia (*p* = 0.01), O&G skills (*p* = 0.015) and dermatology skills (*p* = 0.01). No significant associations were found between overall competence and place of undergraduate training and postgraduate family medicine training.

**TABLE 6:** Association of overall competence in procedural skills and demographic variables.

Demographic variables	Overall competence in procedural skills*									
	Anaesthesia	ENT	Surgery	O&G	Orthopaedics	Medicine	Paediatrics	Ophthalmology	Dermatology	Urology
Age	0.03	0.57	0.86	0.65	0.72	0.96	0.52	0.39	0.53	0.87
Gender	0.66	0.5	0.03	0.41	0.02	0.13	1	0.88	0.18	0.005
Years of experience	0.41	0.97	0.67	0.09	0.84	0.86	0.2	0.78	0.02	0.12
Discipline (where spent most of the time)	0.05	0.85	0.62	0.39	0.24	0.07	0.44	0.54	0.18	0.89
Undergraduate study (in SA or outside SA)	0.77	0.31	0.23	0.34	0.52	0.14	0.35	0.47	0.75	0.44
Family medicine training	0.6	0.3	0.69	0.26	0.59	0.87	0.91	0.6	0.74	0.28
Current hospital	0.01	0.19	0.15	0.015	0.16	0.94	0.78	0.05	0.01	0.26

ENT, ear, nose and throat; O&G, obstetrics and gynaecology; SA, South Africa.

* , *p* values.

## Discussion

When assessing the 72 clinical procedures commonly done in peri-urban district hospitals, the doctors reported varying levels of competence and experience in the different groups of skills. The reported competence levels of doctors also varied according to different disciplines. They considered themselves to be less competent in anaesthesia; ear, nose and throat (ENT) and ophthalmology procedures than general surgery, medicine, orthopaedics, O&G, urology and paediatric procedures. These findings were similar to other studies which identified skill gaps in emergency and anaesthetic procedures in district hospitals.^9,11^ The study findings were different to the other studies done in South Africa where GPs reported higher competence in doing surgical and O&G procedures in urban settings.^14,15^ The competence of doctors in doing procedures varied depending on the type of procedures done and on the context of the hospital as seen in previous studies.^9,10,11,14^

Among the emergency procedures such as cricothyroidotomy and intraosseous infusion, doctors reported less competence in doing these independently; this is reiterated in other studies done in South Africa.^11,27^ Being rare events,^28^ doctors seldom need to perform these procedures in rural or urban settings. However, as these are emergency procedures, ideally all doctors should be able to perform them independently. This emphasises the importance of ongoing doctor training in emergency courses such as Basic Emergency Skills Training or Advanced Trauma Life Support.^27^

Among the anaesthetic procedures, most doctors were competent in performing spinal anaesthesia, while fewer than half of the doctors reported being able to do general anaesthesia independently. The latter finding was not reiterated compared to other studies done in rural settings; this could have been because of the differences in study designs.^9,10,11^ Ability to perform spinal and general anaesthesia is important because, firstly, spinal anaesthesia is a requirement for common procedures such as caesarean sections done at district hospitals. Secondly, it is a clinical imperative that doctors are able to convert a failed spinal anaesthesia to general anaesthesia and take control of the airway and ventilation, preferably by intubation. Thirdly, the Saving Motherhood Initiative identifies inadequate competence in C/S as a major factor contributing to maternal mortality in mothers in district hospitals.^29^All doctors should be trained to have adequate experience in doing C/S and general anaesthesia independently which are core competencies for doctors required at district hospital level.

It is an interesting finding in this study that doctors assessed themselves as not competent in doing simple procedures like IUCD insertion, whereas they are competent in more complicated procedures such as tubal ligation and laparotomy for a ruptured ectopic. This may be because of not practising some of these skills.^15,16,30^ These procedures were not routinely done at district hospital level at the time but mostly done in primary health care (PHC) clinics. Currently, nurse practitioners perform simple procedures such as IUCD insertion in clinics, but if there are complications, generalist doctors in district health services manage them. This suggests that there is a need for rotation of generalist doctors within all levels of the district health system, to ensure appropriate exposure to skills performed at that level.

The data show that the doctors self-reported as being incompetent in procedures such as epidural anaesthesia, appendicectomy, proctoscopy and pleural biopsies, not routinely performed in urban settings. These specialised skills are easily accessible at regional hospitals. These procedures were not done in adequate numbers in rural hospitals of Western Cape either,^14^ and this reiterates the need for context-specific skill sets in urban and rural district hospital settings.

The NHI white paper towards UHC specifies family medicine, O&G, paediatrics and general surgery as the four main domains for district hospitals in South Africa.^3^ This study shows reduced competency in surgical procedures and highlights the need to bridge these gaps in a peri-urban district hospital setting. This will reduce the current over-reliance on higher levels of care, leading to long waiting times and surgical backlogs.^31^

The overall reported competence in the different disciplines did not show any association with the different age groups in this study except for anaesthesia. The overall competence in anaesthetic skills was perceived as higher in the younger age group (25–34 years) than older doctors. The study by De Villiers showed greater self-reported competence in older doctors.^11^ This difference could be because of more clinical exposure of younger doctors to anaesthetic training during the mandatory 2-year internship and community service rotations, introduced in 2005 and 1999, respectively. Alternatively, more senior doctors who did not have mandatory exposure to anaesthetics during internship could be practising in disciplines that do not require anaesthetic skills, especially considering that more than half (57.6%) worked in casualty and OPD at the time of the study.

The gender variations in the overall competency in surgical, orthopaedic and urology skills are significant, with male doctors reported as being more competent in the procedural skills in these disciplines than their female counterparts.^32,33^ This could, in part, be because of the self-reporting nature of the study, where female doctors tend to underestimate confidence in their abilities, especially in terms of competence,^34,35^ and therefore this finding may not be a true reflection of actual competence; if it was, lower workload, lifestyle and income are some of the other contributable factors for these female preferences as described in the literature.^36^ This has implications for current and future human resource for health planning, where women are becoming a significant proportion of the medical workforce.^37^

The self-perceived overall competence in dermatological skills had a significant association with years of experience and this may be because doctors were routinely performing common procedures such as cauterisation, skin biopsies and excision of bumps and lumps.^38^

The overall competence reported on various skill sets did not demonstrate any differences based on undergraduate training, within or outside of South Africa. Given that approximately half of the medical doctors in the study were foreign qualified,^39^ assessing their procedural competencies had to be a consideration for service delivery. It is beyond the scope of this study to determine if the foreign qualified doctors in this study were competent in these procedures prior to or after coming to South Africa, as the majority of these doctors had more than 10 years of working experience in South Africa.

Family medicine training has evolved in South Africa, from the initial part-time Masters in Family Medicine (M Fam Med) programme to the current full-time Masters in Medicine in the branch of Family Medicine (M Med Family Medicine) programme started in 2008. One of the main refinements to the new M Med programme was the subsequent development of a standardised clinical skill set for family medicine registrars.^19^ The doctors in this study exposed to family medicine training were doing the M Fam Med, and there was no difference between their procedural skills and others. The current full-time M Med registrar programme has been running for 10 years now; it is an opportune period to explore and study the procedural skills competencies of these graduates, to determine if, indeed, there is improvement compared to the current revised skills list.^40^ Family physicians have a big role to play in PHC and the long-term goal should be to develop a critical mass of skilled family physicians who can fulfil the key roles of a care provider, supervisor and capacity builder to other cadres of health workers in the district health services.^8^

The study also found that the doctors working in one district hospital showed an overall higher reported competence in doing anaesthesia and O&G procedures than doctors working in the other two hospitals. The central question was ‘What was different in this one hospital?’ This difference may have been because of doctors regularly rotating among the different domains in the one hospital compared to the others, where doctors had worked in the same domains for years. In the opinion of the authors, this element of ‘departmentalisation’ within a district hospital is not ideal in developing generic and cross-cutting procedural skills competencies. Emphasis should be on developing competencies in all the required skills needed for a district hospital level such as in the current family medicine registrar programme.

As this was a self-assessment study, there was potential for information bias, so competencies reported may not have been the actual competence of the participant. Both competence and experience were self-reported and may have been under- or overestimated. The smaller sample size in some of the groups could have affected the power of the study and results. Lastly, this was a cross-sectional study and cannot infer a causal relationship in the associations determined. This design is also a snap shot in time and precludes any inferences regarding longitudinal relationships.

## Conclusion

The self-assessment tool used in this study offered a valuable way of measuring the study objectives and could be used to evaluate the skills of doctors working in district hospitals in other areas or districts in similar settings. This study found variable reported procedural skills competencies among doctors in district hospitals in the southern Gauteng Province, especially in medicine, paediatric, O&G, orthopaedic, surgical and anaesthetic procedures. To the extent that these findings have implications for service delivery and training in the district; interventions aimed at bridging these skill gaps need to take cognisance of variations in sociodemographic and workplace characteristics. There should be provision for generalist doctors and specialists, such as family physicians, to practise a wide range of procedural skills dependent on their experience and competence in a district hospital setting, based on rural–urban contexts and district service delivery packages.
